# Predictors of patient satisfaction with hospital health care

**DOI:** 10.1186/1472-6963-6-102

**Published:** 2006-08-16

**Authors:** José M Quintana, Nerea González, Amaia Bilbao, Felipe Aizpuru, Antonio Escobar, Cristóbal Esteban, José Antonio San-Sebastián, Emilio de-la-Sierra, Andrew Thompson

**Affiliations:** 1 Unidad de Investigación, Hospital de Galdakao, Galdakao, España; 2 Fundación Vasca de Innovación e Investigación Sanitarias (BIOEF), Sondika, España; 3 Unidad de Investigación, Hospital de Txagorritxu, Vitoria-Gasteiz, España; 4 Unidad de Investigación, Hospital de Basurto-Bilbao, Bilbao, España; 5 Servicio de Neumología, Hospital de Galdakao, Galdakao, España; 6 Unidad de Calidad, Hospital de Cruces, Cruces, España; 7 Servicio de Psiquiatría y Psicología, Hospital de Basurto-Bilbao, Bilbao, España; 8 School of Social and Political Studies, University of Edinburgh, Edinburgh, Great Britain

## Abstract

**Background:**

We used a validated inpatient satisfaction questionnaire to evaluate the health care received by patients admitted to several hospitals. This questionnaire was factored into distinct domains, creating a score for each to assist in the analysis.

We evaluated possible predictors of patient satisfaction in relation to socio-demographic variables, history of admission, and survey logistics.

**Methods:**

Cross-sectional study of patients discharged from four acute care general hospitals. Random sample of 650 discharged patients from the medical and surgical wards of each hospital during February and March 2002. A total of 1,910 patients responded to the questionnaire (73.5%). Patient satisfaction was measured by a validated questionnaire with six domains: information, human care, comfort, visiting, intimacy, and cleanliness. Each domain was scored from 0 to 100, with higher scores indicating higher levels of patient satisfaction.

**Results:**

In the univariate analysis, age was related to all domains except visiting; gender to comfort, visiting, and intimacy; level of education to comfort and cleanliness; marital status to information, human care, intimacy, and cleanliness; length of hospital stay to visiting and cleanliness, and previous admissions to human care, comfort, and cleanliness. The timing of the response to the mailing and who completed the questionnaire were related to all variables except visiting and cleanliness. Multivariate analysis confirmed in most cases the previous findings and added additional correlations for level of education (visiting and intimacy) and marital status (comfort and visiting).

**Conclusion:**

These results confirm the varying importance of some socio-demographic variables and length of stay, previous admission, the timing of response to the questionnaire, and who completed the questionnaire on some aspects of patient satisfaction after hospitalization. All these variables should be considered when evaluating patient satisfaction.

## Background

The number of patient satisfaction questionnaires has proliferated over the last decades as tools to measure health care from the patients' perspective [1-3]. One common target group has been patients admitted to a hospital, because admission can be a stressful and dissatisfying experience for many people and because of the high health care costs that an admission to a health care system entails.

As with other measurement instruments, patient satisfaction questionnaires must be tested for validity and reliability [4]. These are basic properties that researchers try to show for their instruments. Beyond these, other possible sources of bias may arise when collected data must be analyzed.

Questionnaires can be completed by different methods: self-reporting, face-to-face interviewing, phone interviewing, or most recently by computer. The self-reporting method requires that the questionnaire is given to the patient at a specific time point, either personally, by mail, or by Internet. Although the Internet may become a frequent way of providing and completing questionnaires [5], in many countries this is either rare or used by a very homogeneous and different group of people from the general population. This explains why mailing is still a frequent method of delivering questionnaires to selected individuals [6]. A major problem and source of bias are patients who do not complete the questionnaire [7]. In order to minimize the number of missing people, researchers typically send reminders, up to two or three, after the first mailing. Additionally, they might contact by phone those who do not respond to try to encourage them to answer the questionnaire, although this is an additional source of bias that has already been studied [8].

Patients admitted to hospitals are generally old and in some cases have different handicaps or functional limitations that prevent or make it difficult for them to complete a questionnaire. For this reason, the interviewed patient may enlist the help of a relative or friend to answer the questionnaire, and this could be a source of bias [9].

In 2002, we used a validated inpatient satisfaction questionnaire to evaluate the health care received by patients admitted to several hospitals [10]. As an advantage over other questionnaires, we had factored it into distinct domains, creating a score for each to assist in the analysis We used a self-reported version of the questionnaire delivered by mail and allowed patients to complete them personally or with the help of a relative or friend, with the stipulation that they indicate who completed it. One of the purposes of this study was to determine and evaluate possible predictors of satisfaction in relation to the more commonly studied socio-demographic variables, as well as the admission history and survey completion logistic variables.

## Methods

### Questionnaire development

Various sources and methods were used to determine the questions to be included in the questionnaire. First, a literature search was undertaken between January and April 2000, using MEDLINE and PSYCLIT databases, that aimed to analyze the instruments that had been devised so far to evaluate inpatient satisfaction at the national and international levels.

Second, eight focus groups were conducted with patients and two with health care professionals to explore opinions about the most positive and negative aspects of care received during the course of a hospital stay. These focus groups were geared towards understanding the issues and recording expressions that could be used to develop questions to be included in the questionnaire.

Thirdly, the research team developed a pool of question items, in relation to the literature and focus groups, to be included in the questionnaire. These items were shown to a group of patients and health professionals, who provided their opinions about the appropriateness of the items and the ability to comprehend them and evaluated the content and face validity of the questions. An initial version of the questionnaire was created, which was evaluated in a pilot study, to analyze the comprehensibility and clarity of the items and features related to the psychometric properties of the instrument. The results of the pilot study led to an amended questionnaire, i.e., the instrument used during the fieldwork described in the current study.

The final questionnaire included 34 questions, which follow in chronologic order the steps from the time the patient is admitted to hospital until discharge [10]. The questionnaire included six domains: information and communication with doctors (12 items), nursing care (8 items), comfort (6 items), visiting (4 items), privacy (2 items), and cleanliness (2 items) (See Additional file 1-Appendix I). The response scale that we used had a varied number of options, ranging from three to six. The scores for each domain were calculated by adding the answers to all the items in each domain. A linear transformation then was carried out, so that the scoring scale for each domain was standardized between 0 and 100, with a score of 100 indicating the highest level of satisfaction. The questionnaire also contained sociodemographic variables, including age, sex, educational level, professional status, and marital status.

### Study participants

The study was conducted among patients admitted to one of four general acute hospitals in the Basque Health Care Service. The hospitals were selected because of their different geographic locations.

Adults 18 years and older were included if they had remained in the hospital longer than 48 hours. Patients admitted to the Neurology Department were excluded because a high percentage had pathologies of the central nervous system (such as cerebrovascular disease) that could hinder or prevent participation in the study. Patients with serious physical or mental pathologies, such as terminal disease and psychosis, which could make the comprehension and completion of the questionnaire difficult, also were excluded, as were patients whose destination after hospital discharge differed from their usual residence, given the difficulties associated with locating them.

All study participants signed a consent form. The study was in compliance with the Helsinki Declaration and the Research Committees of the participant hospitals gave approval to the study.

### Survey

A random sample of 650 patients who had been discharged between February and March 2002 was contacted for each hospital. Two weeks after discharge, the selected patients received the questionnaire with a prepaid return envelope. A cover letter also was attached that explained the reasons for conducting the survey, encouraged their participation, and guaranteed data confidentiality. A follow-up letter was sent to non-responders 2 weeks later. If they still had not returned the questionnaire 15 days after the first reminder, they received a third letter with a new copy of the questionnaire. The response rate obtained using this method was 73.5%.

### Statistical analysis

Descriptive statistics, including frequencies, percentages, means and standard deviations (SDs), were calculated for the socio-demographic variables. For comparisons between respondents and non-respondents on those variables where we had information, we used a Chi-square test for categorical variables and a t-test or the non-parametric Wilcoxon for continuous variables.

In the univariate analysis, we studied the relationships among the selected sociodemographic variables, the history of current and previous admissions, and survey completion logistics variables with the six dimensions of the questionnaire.

We also compared sociodemographic variables, the history of current admissions, and survey completion logistics variables based on the time of the response to the mailing. The Student *t *test, or analysis of variance (ANOVA), with the Scheffe's method for multiple comparisons, or the Kruskal-Wallis test was used for continuous variables, and the Chi-square test or Fisher's exact probability test for categorical variables. Patient satisfaction scores were also compared by time of response to mailing controlling by person who responded, using the analysis of variance (ANOVA), with the Scheffe's method for multiple comparisons, or the Kruskal-Wallis test.

Finally, general linear models were used to analyse differences in satisfaction scores according to time of response to mailing adjusted by the socio-demographic variables. In all cases, we considered the first mail respondents as the reference group.

In all analyses, *P *< 0.05 was considered statistically significant. All statistical analyses were performed using SAS for Windows statistical software, version 8.0.

## Results

The mean age of the participants was 62.2 years old; 54.3% were men, 68.3% were married or cohabitating, and 54.1% had only a primary education level. Of the 73.5% that participated in the study, 52.4% responded to the first mailing, 27.2% to the first reminder, and 20.4% to the second reminder (Table 1). Those who did not respond were a mean of 63 years old and 48.3% were women, but these differences were not significantly different. We found non-respondents significantly more likely to be emergency admissions in medical, rather than surgical, specialties, discharged to a place other than their normal residence, and a with a higher mean length of stay The univariate analysis of the selected variables showed their relationship with the scores of our satisfaction questionnaire (Table 2). We found that age was statistically correlated with all domains except the visiting domains, with higher satisfaction scores related to increasing age. Gender was related to comfort, visiting and intimacy, with men expressing higher satisfaction. Level of education was only related to comfort and cleanliness, with higher satisfaction among those with no schooling or a primary education level of studies. Marital status was correlated with the information, human care, intimacy, and cleanliness domains, with those married or cohabitating having higher scores except for the cleanliness domain. Shorter length of stay showed more satisfaction with visiting and cleanliness. Those admitted previously showed lower satisfaction with human care, comfort and cleanliness. Patients who responded to the 1^st ^mailing expressed higher satisfaction than those who responded to the 2^nd ^or 3^rd ^mailings, with Information, Human Care, Comfort and Intimacy. Finally, the variable "who responds to the questionnaire" revealed that those who responded alone expressed higher satisfaction with information, human care, comfort and intimacy.

We checked whether there were differences by time of response to the mailing for different variables (Table 3) and we found this was significant for age, marital status and who completed the questionnaire; i.e. later response was more likely by older patients, widowed and those who needed help to answer the questionnaire. There were no discernible differences by gender, level of studies, or working status.

When evaluating the questionnaire satisfaction scores based on the time of response to the mailings and who responded to them, we observed that those who completed the questionnaire with help systematically had lower scores than those who responded themselves (Table 4). Statistically significant differences differences were found in the three mailings and for the information, human care, comfort, and intimacy domains but not for the visiting and cleanliness domains either for those who responded themselves or with help. A trend toward higher satisfaction was found for those who responded early and a trend toward lower satisfaction was found for those who responded later for those who responded themselves or with help.

We also studied the effect of the previous variables on the satisfaction scores after adjustment by all variables (Table 5) in a multivariate model. Age was significantly related to all domains with higher scores increasing with age. Gender showed similar results as in the univariate analyses. The level of education was related to visiting, intimacy, and cleanliness, with those with no education or primary studies having higher scores. Marital status was related to information, with higher scores for married patients; single or divorced individuals had higher scores for comfort, visiting, and cleanliness. Length of stay related to comfort, visiting and cleanliness, with lower scores for those with longer length of stay. Previous admissions related to information, human care, comfort, visiting and cleanliness, with lower scores for those with more than four previous admissions. Lower scores were also found for respondents to the 2^nd ^and 3^rd ^mailings, compared with the 1^st ^mailing, for Information and Human Care, and specifically from the 3^rd ^mailing compared with the 1^st ^mailing for Comfort, Intimacy and Cleanliness. In all cases, a decrease in the scores is seen from the 2^nd ^to the 3^rd ^mailing. Finally, those who responded with help had lower scores on information, human care, comfort, intimacy and cleanliness. The R^2 ^of each domain model also was estimated, and ranged between 0.028 (visiting) to 0.084 (comfort).

## Discussion

Our study showed that age, education level, marital status, sex, work status, length of stays, and previous admissions affected the scores of the six domains in the satisfaction questionnaire. Some variables had been studied previously, but to the best of our knowledge, the full range of variables included in this article had not been studied together in multivariate analyses for the different domains of a validated satisfaction questionnaire and with a large sample size. The validity of our satisfaction questionnaire was discussed previously and showed acceptable results. Nevertheless, to avoid bias we outlined two methodologic aspects: the reminders needed before a patient answered the questionnaire and if the patient completed the questionnaire or did so with the assistance of someone else.

As in previous studies, we showed that older patients tended to have higher satisfaction scores in all areas of our questionnaire [11,12]. Similarly, those with no education or only primary education had higher satisfaction scores. Marital status traditionally has been included in this kind of study [13], and usually, those married or cohabitating tended to have higher satisfaction scores, but in our study those who were single or divorced had higher satisfaction scores in the comfort, visiting, and cleanliness domains. In contrast to other studies [11], our results showed that men tended to have higher satisfaction scores than women, as in others [14,15]. We also studied the influence of the working status of our respondents but, unlike others [16], we did not find that this variable had any influence on our sample patient satisfaction. This can be due, in our case, to the fact that there was little variability since most male respondents had retired and most women were working at home. We did not evaluate if the type of insurance had any influence since 100% of our patients were covered by the public National Health System.

The longer the length of stay of the index admission studied, the lower the satisfaction on specific domains such as comfort, visiting, and cleanliness, which seemed logical, as in other studies [14]. However, studies of mental health services have found the opposite to be true [17], and those seem to be areas more likely conditioned by a long stay. In addition, we found that patients who already had had a previous hospital admission tended to be more demanding or critical and have lower satisfaction levels on relevant areas such as information or human care, comfort, visiting, or cleanliness.

Two main aspects of the logistics of a self-administered patient questionnaire were studied here: the effect of the time when the patient responded to our mailings, with those who responded to a second or third reminder having lower levels of satisfaction; and who complete the questionnaire, the patient or someone else. The latter had the worse scores on all domains of the satisfaction questionnaire, except for the visiting domain. These logistic aspects also have been studied previously[6,8] not finding the later differences among delay on response and patients' satisfaction, but this could be due to the small sample size included in that study compared to ours (between 78 to 254 patients depending on the time when the patient responded to their mailings), little lower response rate, the design of the study, and the use of different satisfaction questions, though they found a tendency similar to ours in the decline in their satisfaction levels. But so many determinants of the satisfaction of hospitalized patients have not been studied globally together in multivariate models. The current study provides valuable information on the effect of all variables on the different domains that constitute our patient satisfaction tool. Therefore, we provide a more complete picture of the determinants of the satisfaction of the various domains.

The different studies that have evaluated the effect of the previous sociodemographic variables [13], previous admission experience [18], the length of hospitalization [14], and survey logistics showed, in some cases, contradictory results.

Studies of the impact of reminders on the results of patient satisfaction surveys are contradictory: some have shown that reminders affect the patients' responses [19], as in our study, while others did not show that effect [8].

Patient satisfaction can be measured in different ways. Among them, the use of surveys has been a common way of conducting patient satisfaction studies. The forms of administration of the survey, that is, self-administration, personnel interview, or a phone interview, have been evaluated in different studies[6,20]. However, the effect of who completed the questionnaire has been studied less often. We showed in the current study that who completed the questionnaire has an important effect on the results, in that a more negative satisfaction level was recorded on those surveys answered by someone other than the patient.

The limitations of this study include the type of design chosen (descriptive) and the inevitable non-responders. As in previous studies [21,22], the R^2 ^values were low, indicating room for improving the prediction of patient satisfaction with other variables not included here. In addition, the range of possible explicative variables included in this study, although large, was not as exhaustive as we would have wished. Several other variables have been evaluated previously that also showed a relationship with patient satisfaction, as the previous health status or hospital characteristics[22]. However, we would theoretically expect most explanation to be given by the differences in health care experiences, rather than the characteristics of the patients and their method of responding to a survey.

## Conclusion

We concluded that, as in previous studies, there is evidence that patient sociodemographic characteristics affect patient satisfaction levels. In addition, it is logical that previous admissions and the length of the current admission also affect the patient response. Also, depending on the manner in which the survey was administered, the mail interview method may obtain high response rates when using reminders, but those reminders affect the patient responses. Finally, we must consider who completed the questionnaire, the patient or someone else. Therefore, when conducting a patient satisfaction survey we must be aware of the effect of many variables on the patient responses and make the appropriate adjustments to provide valid results.

## Competing interests

The author(s) declare that they have no competing interests.

## Authors' contributions

JMQ and NG conceived of the study, coordinated and participated in the design of the study, and drafted the manuscript. AE, FA, CE, JASS, ES and AT participated in the design and helped to draft the manuscript. AB, AT performed the statistical analyses and drafted the manuscript. All authors read and approved the final manuscript.

## Pre-publication history

The pre-publication history for this paper can be accessed here:



## Supplementary Material

Additional file 1 Summary of the questions included in the patient satisfaction questionnaire. Short description of the data: questions included in the questionnaire, for each of the six domains that were obtained in the validation study; these questions have been summarized and listed in the file. Click here for file

## Figures and Tables

**Table 1:** Sociodemographic data of the total sample

Variables	Responders n = 1910		Non-Responders n = 703		*P* value
	
	n	%	n	%	
Age (years) ± SD	62.2	16.6	63.0	19.1	0.35
Length of stay (days) x¯ MathType@MTEF@5@5@+=feaafiart1ev1aaatCvAUfKttLearuWrP9MDH5MBPbIqV92AaeXatLxBI9gBaebbnrfifHhDYfgasaacH8akY=wiFfYdH8Gipec8Eeeu0xXdbba9frFj0=OqFfea0dXdd9vqai=hGuQ8kuc9pgc9s8qqaq=dirpe0xb9q8qiLsFr0=vr0=vr0dc8meaabaqaciaacaGaaeqabaqabeGadaaakeaacuWG4baEgaqeaaaa@2E3D@ ± SD	7.6	5.9	8.5	7.4	<0.05
Sex					0.23
Men	1,026	54.3	361	51.7	
Women	862	45.7	337	48.3	
Admission service					<0.001
Medical	1,084	56.8	452	64.6	
Surgical	826	43.2	248	35.4	
Admission type					<0.001
Urgent	755	62.4	320	78.8	
Planned	456	37.6	86	21.2	
Marital status					
Single	232	12.6			
Married/cohabitating	1,261	68.3			
Separated/divorced	48	2.6			
Widowed	304	16.5			
Education level					
No education	288	15.9			
Primary studies	982	54.1			
High school/secondary education	395	21.8			
University	148	8.2			
Occupation					
Employed full time	330	18.8			
Employed part time	58	3.3			
Homemaker	313	17.8			
Unemployed	56	3.2			
Student	19	1.1			
Retired	825	47.0			
Disabled	155	8.8			
Time of response*					
1^st ^mailing	1,001	52.4			
2^nd ^mailing	520	27.2			
3^rd ^mailing	389	20.4			
Previous admission					
Yes	1,509	82.1			
No	330	17.9			
No. previous admissions					
<= 2	542	39.3			
3–4	437	31.7			
>4	400	29.0			
Who completed the questionnaire					
Patient	1026	55.6			
Someone else	818	44.4			

The sample size in each question differs because not all subjects answered all questionnaire items.

*The response rate is calculated on the basis of the total number of questionnaires returned.

Frequencies and percentages are presented, except for age and length of hospital stay, where the means and SDs are presented.

**Table 2:** Univariate analysis by relevant variable

Variables	Patient satisfaction questionnaire domains					
	
	Information	Human Care	Comfort	Visiting	Intimacy	Cleanliness
	
	x¯ MathType@MTEF@5@5@+=feaafiart1ev1aaatCvAUfKttLearuWrP9MDH5MBPbIqV92AaeXatLxBI9gBaebbnrfifHhDYfgasaacH8akY=wiFfYdH8Gipec8Eeeu0xXdbba9frFj0=OqFfea0dXdd9vqai=hGuQ8kuc9pgc9s8qqaq=dirpe0xb9q8qiLsFr0=vr0=vr0dc8meaabaqaciaacaGaaeqabaqabeGadaaakeaacuWG4baEgaqeaaaa@2E3D@ (SD)	x¯ MathType@MTEF@5@5@+=feaafiart1ev1aaatCvAUfKttLearuWrP9MDH5MBPbIqV92AaeXatLxBI9gBaebbnrfifHhDYfgasaacH8akY=wiFfYdH8Gipec8Eeeu0xXdbba9frFj0=OqFfea0dXdd9vqai=hGuQ8kuc9pgc9s8qqaq=dirpe0xb9q8qiLsFr0=vr0=vr0dc8meaabaqaciaacaGaaeqabaqabeGadaaakeaacuWG4baEgaqeaaaa@2E3D@ (SD)	x¯ MathType@MTEF@5@5@+=feaafiart1ev1aaatCvAUfKttLearuWrP9MDH5MBPbIqV92AaeXatLxBI9gBaebbnrfifHhDYfgasaacH8akY=wiFfYdH8Gipec8Eeeu0xXdbba9frFj0=OqFfea0dXdd9vqai=hGuQ8kuc9pgc9s8qqaq=dirpe0xb9q8qiLsFr0=vr0=vr0dc8meaabaqaciaacaGaaeqabaqabeGadaaakeaacuWG4baEgaqeaaaa@2E3D@ (SD)	x¯ MathType@MTEF@5@5@+=feaafiart1ev1aaatCvAUfKttLearuWrP9MDH5MBPbIqV92AaeXatLxBI9gBaebbnrfifHhDYfgasaacH8akY=wiFfYdH8Gipec8Eeeu0xXdbba9frFj0=OqFfea0dXdd9vqai=hGuQ8kuc9pgc9s8qqaq=dirpe0xb9q8qiLsFr0=vr0=vr0dc8meaabaqaciaacaGaaeqabaqabeGadaaakeaacuWG4baEgaqeaaaa@2E3D@ (SD)	x¯ MathType@MTEF@5@5@+=feaafiart1ev1aaatCvAUfKttLearuWrP9MDH5MBPbIqV92AaeXatLxBI9gBaebbnrfifHhDYfgasaacH8akY=wiFfYdH8Gipec8Eeeu0xXdbba9frFj0=OqFfea0dXdd9vqai=hGuQ8kuc9pgc9s8qqaq=dirpe0xb9q8qiLsFr0=vr0=vr0dc8meaabaqaciaacaGaaeqabaqabeGadaaakeaacuWG4baEgaqeaaaa@2E3D@ (SD)	x¯ MathType@MTEF@5@5@+=feaafiart1ev1aaatCvAUfKttLearuWrP9MDH5MBPbIqV92AaeXatLxBI9gBaebbnrfifHhDYfgasaacH8akY=wiFfYdH8Gipec8Eeeu0xXdbba9frFj0=OqFfea0dXdd9vqai=hGuQ8kuc9pgc9s8qqaq=dirpe0xb9q8qiLsFr0=vr0=vr0dc8meaabaqaciaacaGaaeqabaqabeGadaaakeaacuWG4baEgaqeaaaa@2E3D@ (SD)
Age (years)						
<= 50	77.3 (19.8)	75 (21.3)	61.4 (17.7)	77.9 (20.3)	88 (21.4)	87.1 (17.7)
51–65	83.9 (17.7)	80.2 (20.2)	66 (18.5)	79.7 (18.7)	91.8 (18.4)	88.7 (17.4)
>65	82.3 (18.2)	78.8 (20.9)	69.5 (17.7)	80.2 (18)	90.9 (20.1)	90 (17.1)
*P *value	<0.001	<0.001	<0.001	0.31	<0.01	<0.01
Gender						
Men	81.5 (18.9)	78.6 (20.7)	67.9 (18.2)	80.6 (18.1)	92.4 (18.1)	89.6 (16.8)
Women	81.5 (18.3)	77.7 (21)	65.2 (18)	78.3 (19.5)	88 (22.1)	88.2 (18)
*P *value	0.52	0.29	<0.001	<0.01	<0.001	0.09
Education level						
No studies/primary	81.8 (18.1)	78.4 (20.8)	67.3 (17.6)	80.2 (17.9)	90.7 (19.6)	89.5 (16.9)
Secondary/university	80.8 (19.6)	78.2 (20.8)	64.8 (18.8)	78.1 (20.3)	89.6 (21.4)	87.8 (17.8)
*P *value	0.80	0.80	<0.01	0.25	0.57	<0.05
Marital status						
Single/divorced	77.9 (20.5)	75.9 (22.6)	66.6 (17.5)	80.7 (18.9)	88.0 (21.5)	91.0 (16.5)
Widowed	80.7 (18.0)	76.4 (21.1)	66.1 (18.4)	78.6 (18.6)	89.1 (21.4)	88.3 (18.4)
Married/cohabitating	82.4 (18.3)	79.1 (20.4)	66.6 (18.2)	79.4 (18.8)	91.3 (19.3)	88.6 (17.3)
*P *value	<0.001	<0.05	0.79	0.11	<0.05	<0.05
Length of hospital stay (days)						
<4	81.8 (18.5)	78.9 (20.2)	67.4 (18.6)	82.2 (17.8)	90.2 (20.4)	91.1 (15.4)
4–7	81.5 (18.3)	78.5 (20.6)	66.7 (18.2)	79.6 (18.7)	91.4 (19.3)	88.8 (17.5)
>7	81.6 (18.8)	77.7 (21.6)	66.3 (18.1)	77.9 (19.2)	90.0 (20.3)	87.9 (18.2)
*P *value	0.89	0.84	0.47	<0.001	0.32	<0.05
Previous admissions						
0	82.1 (17.0)	81.0 (18.8)	68.2 (18.1)	80.5 (18.5)	89.5 (20.8)	90.9 (16.0)
1–4	81.5 (19.3)	78.8 (20.5)	66.7 (18.0)	80.0 (18.6)	90.7 (20.1)	88.9 (17.3)
>4	79.9 (18.5)	75.1 (21.5)	64.3 (17.7)	78.6 (19.3)	90.6 (19.4)	87.1 (18.3)
*P *value	0.07	<0.001	<0.01	0.23	0.51	<0.01
Response to mailing						
1^st ^mailing	83.4 (17.6)	80.9 (19.9)	68.0 (18.6)	79.3 (19.0)	91.8 (18.6)	89.4 (16.6)
2^nd ^mailing	80.9 (19.4)	77.6 (21.1)	67.3 (17.5)	79.8 (18.0)	90.3 (19.6)	89.3 (17.6)
3^rd ^mailing	77.8 (19.2)	72.4 (21.9)	62.6 (17.9)	79.8 (19.2)	87.8 (23.1)	87.5 (18.8)
*P *value	<0.001	<0.001	<0.001	0.71	<0.05	0.34
Responded to questionnaire						
With help	79 (19.1)	75.8 (21.3)	65.7 (17.4)	80 (18.1)	88.6 (21.3)	88.4 (17.8)
Alone	83.4 (18.1)	80.2 (20.4)	67.5 (18.6)	79.1 (19.3)	92 (18.8)	89.4 (17)
*P *value	<0.001	<0.001	<0.05	0.85	<0.001	0.28

Student *t *test, or analysis of variance (ANOVA) with the Scheffe's method for multiple comparisons, or the Kruskal-Wallis test performed.

**Table 3:** Descriptive statistics by mailing

Variable	Time of response to mailing			
		
	1^st ^mailing n = 1001	2^nd ^mailing n = 520	3^rd ^mailing n = 389	*P *value
Age (years)				<0.05
≤50	239 (24.0)	95 (18.5)	97 (25.1)	
51–65	261 (26.2)	146 (28.4)	87 (22.5)	
>65	496 (49.8)	274 (53.2)	202 (52.3)	
Gender				0.53
Men	532 (53.6)	289 (56.5)	205 (53.4)	
Women	460 (46.4)	223 (43.6)	179 (46.6)	
Education level				<0.05
None/primary school	642 (67.3)	358 (73.2)	270 (73.0)	
Secondary/university	312 (32.7)	131 (26.8)	100 (27.0)	
Work status				0.11
Working/homemaker	382 (41.4)	169 (36.1)	150 (41.1)	
Unemployed	34 (3.7)	11 (2.4)	11 (3.0)	
Retired/disabled	499 (54.1)	279 (59.6)	202 (55.3)	
Student	8 (0.9)	9 (1.9)	2 (0.6)	
Marital status				<0.01
Married/cohabitating	693 (71.5)	333 (66.9)	235 (62.2)	
Single/divorced	142 (14.7)	78 (15.7)	60 (15.9)	
Widowed	134 (13.8)	87 (17.5)	83 (22.0)	
Who fulfill the questionnaire				<0.001
Alone	586 (60.6)	258 (51.8)	182 (48.0)	
With help	381 (39.4)	240 (48.2)	197 (52.0)	

Data are given as frequency (percentage).

**Table 4:** Mean patient satisfaction scores by response to mailing times and person who responds

	Time of response to mailing			
	
Variables	1^st ^mail n = 967	2^nd ^mail n = 498	3^rd ^mail n = 379	*P *value
	n = 586	n = 258	n = 182	
Responded alone				
Information	84.9 (17.1)	82.4 (20.0)	80.2 (17.5)	<0.01
Human care	82.3 (19.4)	79.5 (20.5)	74.5 (21.9)	<0.001
Comfort	69.1 (18.9)	68.3 (17.1)	61.3 (18.4)	<0.001
Visit	78.8 (19.8)	79.2 (17.9)	79.9 (19.6)	0.46
Intimacy	92.9 (17.5)	91.3 (19.7)	90.3 (21.4)	0.48
Cleanliness	89.5 (16.7)	90.9 (14.9)	86.8 (20.3)	0.22
				
Responded with help	n = 381	n = 240	n = 197	
Information	80.8 (18.3)	79.4 (18.9)	75.0 (20.3)	<0.01
Human care	78.6 (20.6)	76.1 (21.1)	70.0 (21.8)	<0.001
Comfort	66.3 (17.8)	66.8 (16.9)	63.3 (16.9)	0.07
Visit	80.2 (17.4)	80.3 (18.4)	79.1 (19.0)	0.84
Intimacy	90.1 (20.2)	89.1 (19.6)	85.4 (24.6)	0.10
Cleanliness	89.1 (16.4)	87.7 (20.1)	87.9 (17.5)	0.80

The total number of patients does not match previous tables since there was missing information for those who responded to the "response with/out help" variable.

**Table 5:** Multivariate analysis by relevant variables

Variables	Patient satisfaction questionnaire domains											
	
	Information		Human Care		Comfort		Visiting		Intimacy		Cleanliness	
	
	β Coef.	*P *value	β Coef.	*P *value	β Coef.	*P *value	β Coef.	*P *value	β Coef.	*P *value	β Coef.	*P *value
R^2^	0.063		0.062		0.084		0.028		0.039		0.034	
Age (years)												
<= 50	-----	-----	-----	-----	-----	-----	-----	-----	-----	-----	-----	-----
51–65	7.10	<0.001	6.35	<0.001	6.30	<0.001	2.52	0.08	3.95	<0.05	3.21	<0.05
>65	7.66	<0.001	8.18	<0.001	11.65	<0.001	4.07	<0.01	3.54	0.06	4.99	<0.001
Gender												
Men vs. women	-1.19	0.23	-0.58	0.58	1.91	<0.05	2.01	<0.05	3.44	<0.01	1.34	0.14
Education level												
No studies/primary	-----	-----	-----	-----	-----	-----	-----	-----	-----	-----	-----	-----
Secondary/university	-1.67	0.15	-1.13	0.37	-1.90	0.08	-2.85	<0.05	-2.87	<0.05	-2.31	<0.05
Marital status												
Single/divorced	-2.85	<0.05	-0.96	0.53	3.95	<0.01	3.38	<0.05	-1.64	0.35	4.37	<0.001
Widowed	-0.67	0.64	-1.37	0.37	0.003	0.99	-0.75	0.60	0.48	0.78	1.25	0.34
Married/cohabitating	-----	-----	-----	-----	-----	-----	-----	-----	-----	-----	-----	-----
Length of hospital stay (days)												
<4	-----	-----	-----	-----	-----	-----	-----	-----	-----	-----	-----	-----
4–7	-0.77	0.53	-0.42	0.75	-1.56	0.16	-3.42	<0.01	0.88	0.57	-2.81	<0.05
>7	-0.27	0.82	-1.22	0.36	-2.33	<0.05	-5.50	<0.001	-0.35	0.82	-3.82	<0.001
Previous admissions												
0	-----	-----	-----	-----	-----	-----	-----	-----	-----	-----	-----	-----
1–4	-1.53	0.22	-2.78	<0.05	-2.40	<0.05	-1.35	0.28	0.21	0.89	-2.60	<0.05
>4	-3.3	<0.05	-6.72	<0.001	-5.16	<0.001	-2.92	<0.05	0.10	0.96	-4.41	<0.01
Response to mailing												
1^st ^mailing	-----	-----	-----	-----	-----	-----	-----	-----	-----	-----	-----	-----
2^nd ^mailing	-2.35	<0.05	-3.10	<0.01	-1.32	0.19	-0.03	0.98	-1.57	0.25	-0.08	0.93
3^rd ^mailing	-4.34	<0.001	-6.66	<0.001	-5.39	<0.001	1.06	0.38	-4.10	<0.01	-1.75	0.12
Responded to questionnaire												
With help vs. alone	-7.37	<0.001	-6.31	<0.001	-4.45	<0.001	0.02	0.98	-5.57	<0.001	-2.20	<0.05

β Coef.: Beta coefficient from the lineal general model, after adjustment by all relevant variables. Positive values indicate more satisfaction on that domain for that category; negative values indicate less satisfaction compared with the reference category, which is blank or indicated as "versus".
