# Supplementary material for: Predictors of patient satisfaction with hospital health care
Source: BMC Health Serv Res. 2006 Aug 16;6:102. doi: 10.1186/1472-6963-6-102 (PMC1579213; doi:10.1186/1472-6963-6-102)
Supplement: Additional file 1 — Summary of the questions included in the patient satisfaction questionnaire. Short description of the data: questions included in the questionnaire, for each of the six domains that were obtained in the validation study; these questions have been summarized and listed in the file. [file 1472-6963-6-102-S1.doc]

**Appendix I**

**Summary of the questions included in the patient satisfaction questionnaire**

**INFORMATION & MEDICAL CARE**

- Doctors' explanations of the disease
- Doctors' interest in patients' questions
- Doctors' care of patient
- Global assessment of the information
- Explanation at discharge
- Doctors' care
- Patients' need to ask for information about the health condition
- Doctors' professional training
- Patients' opinion of doctors' explanations
- Discharge report
- Explanations about treatment
- Doctors' use of technical words

**NURSING CARE**

- Nurses' care
- Nurses' empathy
- Nurses' care of patients
- Nurses' professional training
- Nurses' interest in patients' questions
- Global assessment of the care
- Contradictory orders
- Nurses' explanations of the disease

**COMFORT**

- Room conditions for the patient
- Global assessment of the physical conditions of the hospital
- Quality of the food
- Room comfort for the family
- Sleep disturbance due to environmental conditions
- Hour patients were awakened

**VISITING**

- Visitors disturbed by staff
- Visiting hours
- Time the visitors spent in the room
- Quantity of visitors

**PRIVACY**

- Privacy during examination or tests
- Privacy on the way to testing

**CLEANLINESS**

- Toilet cleanliness
- Room cleanliness
